# Proton pump inhibitor use and risk of stroke: A systematic review and meta-analysis

**DOI:** 10.12669/pjms.40.10.10409

**Published:** 2024-11

**Authors:** Yaoyao Feng, Ying Zhong

**Affiliations:** 1 Yaoyao Feng Department of Neurology, Huzhou Third Municipal Hospital, The Affiliated Hospital of Huzhou University, Huzhou, Zhejiang Province 313000, P.R. China; 2 Ying Zhong Department of Geriatrics, Huzhou Third Municipal Hospital, The Affiliated Hospital of Huzhou University, Huzhou, Zhejiang Province 313000, P.R. China

**Keywords:** Proton pump inhibitor, Propensity score matching, Stroke, Systematic review

## Abstract

**Objective:**

To explore a link between the use of proton pump inhibitor (PPI) and the risk of stroke.

**Methods:**

Comprehensive literature search in PubMed, EMBASE, and Cochrane CENTRAL Library databases was carried out for observational studies establishing the link between PPI and a risk of stroke. Data extraction and quality assessment were performed by two reviewers. Pooled hazard ratios (HRs) with 95% confidence intervals (CIs) using random-effects models were plotted. Subgroup analyses were conducted based on age, gender, PPI type, duration of follow-up, and propensity score matching (PSM).

**Results:**

The analysis included 12 studies, with considerable heterogeneity (I2 = 95%). PPI use did not affect the incidence of ischemic stroke (HR: 1.11, 95% CI: 0.98-1.26). Subgroup analyses revealed that PPI use correlated with the risk of ischemic stroke, in particular in patients<65 years old (HR: 1.25, 95% CI: 1.07-1.45), both males (HR: 1.12, 95% CI: 1.02-1.24) and females (HR: 1.21, 95% CI: 1.10-1.33). The correlation varied depending on the PPI type, with pantoprazole showing elevated risk (HR: 1.66, 95% CI: 1.43-1.93). Duration of follow-up or propensity score matching (PSM) did not impact the association.

**Conclusion:**

PPI use may be linked with ischemic stroke, particularly in individuals <65 years old and of all genders. The specific PPI type may also influence the risk. However, the cumulative analysis did not find any statistically significant association, and heterogeneity among studies was substantial.

## INTRODUCTION

PPIs are a class of medications widely prescribed for the management of various gastrointestinal tract disorders,^1^,^2^ and are highly effective in reducing gastric acid secretion by irreversibly binding to the proton pump in the parietal cells of the stomach.^3^,^4^ The efficacy and safety profile of PPIs have contributed to their widespread acceptance and extensive use in clinical practice.^5^,^6^ However, recent studies found that the long-term PPI use may lead to certain adverse outcomes, such as increased risk of stroke.^7^,^8^

Stroke is considered a major cause of mortality and morbidity,^9^-^11^ and is caused by interrupted or diminished blood supply to a part of the brain, leading to neurological deficits. Ischemic stroke results from a blockage or narrowing of the blood vessels supplying the brain, accounts for the majority of stroke cases.^12^ Haemorrhagic stroke, on the other hand, is caused by the bleeding into the brain tissue or surrounding spaces.^13^ Understanding the risk factors of stroke is crucial both for preventing the event and for improving patient outcomes.

Numerous studies have focused on the possible association of the risk of stroke with long term PPI use, with conflicting results.^14^,^15^ While some studies have reported such association, others have failed to establish a significant link.^15^-^17^ A meta-analysis by Nolde et al.^18^ has examined the link between PPI use and stroke. However, it only included five studies. Recently, new reports were published on the matter, making it necessary to update current evidence.

This systematic review aimed to summarize the findings from all relevant studies investigating the link between PPI use and the risk of stroke and identify potential sources of heterogeneity, including different study designs, types of PPIs, and follow-up.

## METHODS

Preferred Reporting Items for Systematic Reviews and Meta-Analyses (PRISMA) guidelines^19^ was followed to ensure transparent and comprehensive reporting of the study findings. The protocol of this study was drafted and uploaded at PROSPERO with registration number CRD42023422247.

### Study Selection:

Embase, PubMed, and Cochrane Library were used to identify possible studies published up until May 2023. The search strategy involved the use of appropriate keywords and MeSH terms related to proton pump inhibitors, stroke, and observational study designs. The search string employed is as follows: (Proton Pump Inhibitors [Mesh] OR Proton Pump Inhibitors OR PPI OR Omeprazole OR Lansoprazole OR Pantoprazole OR Esomeprazole OR Rabeprazole) AND (Stroke [Mesh] OR Stroke OR Cerebrovascular Disorders OR Cerebrovascular Accident OR Cerebrovascular Apoplexy OR Cerebrovascular Insult). Bibliography of relevant articles, conference proceedings and archived issues of relevant journals was screened for any potential eligible studies.

### Inclusion Criteria:

- Cohort studies and case-control studies.
- Studies that examine the connection between PPI use and the incidence rate of stroke.
- Studies reporting HRs with their corresponding 95% CIs, or providing sufficient data to calculate these measures.
- Studies conducted in human populations with no history of cardiovascular events.

### Exclusion Criteria:

- Animal studies.
- Review articles, editorials, commentaries, and letters.
- Studies that do not report measures of link or provide sufficient data to calculate them.
- Non-English language publications.
- Studies not specifically reporting on the link between PPI use and stroke.

### Data Extraction and Quality Assessment:

Initial screening was done by two independent reviewers. Full-texts were then assessed based on the predefined criteria. Data extraction was conducted independently by the reviewers, and included study design, sample size, follow-up duration, participant characteristics (e.g., age, gender), details of PPI exposure (e.g., duration, frequency), stroke outcome assessment (e.g., stroke definition, subtype), and effect estimates (e.g., RRs, ORs, HRs) with corresponding 95% CIs or data necessary to calculate them.

### Quality of included studies:

Quality of the included studies and a risk of bias were assessed by the Newcastle-Ottawa Scale^20^ for cohort studies, and the Newcastle-Ottawa Scale for case-control studies. These tools assessed key aspects such as study design, sample representativeness, outcome ascertainment, exposure assessment, and control of confounding variables.

### Statistical Analysis:

Pooled effect size was calculated by meta-analysis to assess the overall link between PPI use and the risk of stroke. Pooled hazard ratios (HRs) were plotted using random effect models. When necessary, data were transformed to ensure consistency across studies. Heterogeneity was assessed by I² statistic, and subgroup analyses were done to identify potential sources of heterogeneity, such as different study designs, types of PPIs, and stroke subtypes. Sensitivity analyses were performed by excluding studies with high risk of bias or low-quality scores. Funnel plots assessed the publication bias.

## RESULTS

**Fig.1:**
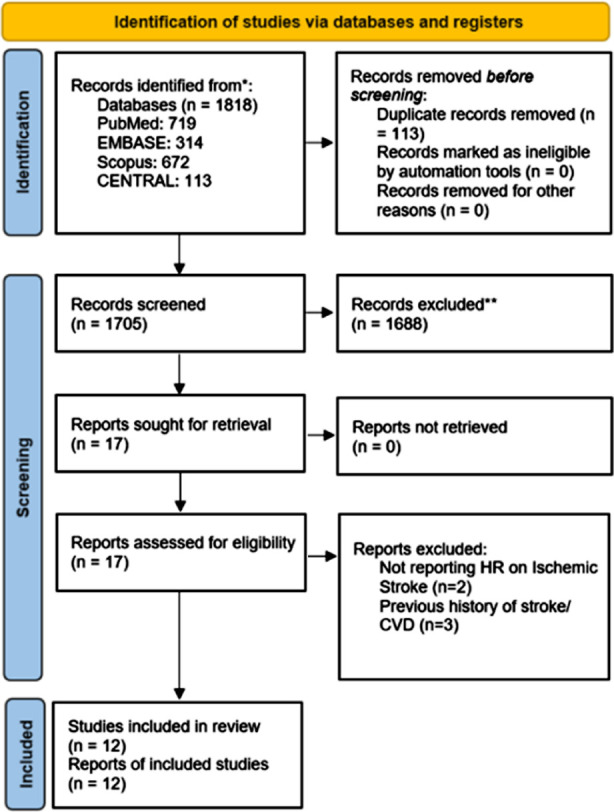
PRISMA flow chart showing the study selection process.

As shown on the detailed PRISMA flow chart (Fig.1), 1818 studies were identified by the literature search, and 12 publications met eligibility criteria for inclusion in the final analysis.^15^-^17^,^21^-^29^ Of them, eight studies were retrospective and four studies were prospective cohorts. The studies were done in China, Korea, Germany, the United States, Israel, Denmark, and Taiwan, involved patients with no previous history of CVDs and varied in terms of PPIs used, comparators, and median follow-up durations, ranging from one year to 12.5 years. Different methods were used to assess stroke occurrence, including incident stroke, brain imaging (CT or MRI), and hospital discharge diagnosis. The studies employed various methods to identify PPI use, such as previous prescriptions, verbal interviews with questionnaires, and self-reported data. This variation in assessment methods provides a comprehensive evaluation of stroke outcomes. The detailed characteristics of all the included studies are shown in Table-I. Quality assessment of the studies is summarized in Table-II.

**Table-I:** Characteristic of included studies.

Author	Year	Country	Study Design	Propensity score matching	Participants	Number of PPI users	Age	Male %	Method of stroke assessment	Method of identification of PPI use	Adjusted Factors	PPI used	Frequency of PPI use	Median Follow-up
Kim et al.	2022	Korea	RS	Yes	8007	5714	20-94	37.60%	NR	NR	None	Pantoprazole, Rabeprazole	NR	10 years
Ma et al.	2022	China	PS	No	13503	1630	60-75 (68.1)	41.60%	Incident stroke	Verbal interview with touchscreen questionaire	Age, sex, race, household income, education level,family history of heart disease and stroke, medication use (NSAID, SSRI, hypoglycemic drugs, statins, PPIs, H2RAs)	All PPIs	Regular use	12.5 years
Park et al.	2022	Korea	RS	No	8445	323	55-75 (66.5)	57.40%	Brain computed tomography or magnetic resonance imaging during admission	Previous prescriptions	Age, household income, hypertension, diabetes mellitus, total cholesterol, body mass index, alcohol consumption, physical activity, smoking habit, proton pump	All PPIs	Regular use	1 year
Bell et al.	2021	United States	PS	No	4346	680	70-80 (75)	42%	Brain computed tomography or magnetic resonance imaging during admission	Direct visual inspection of pill bottles, which could include over-the-counter and prescription medications	Age, sex, race, diabetes, smoking status, systolic blood pressure, antihypertensive medication use, total cholesterol, and high-density lipoprotein cholesterol	All PPIs	NR	5.1 years
Geng et al.	2021	China	RS	Yes	19229	1671	53-70 (60.3)	51%	self-reported data, primary care data, hospital admission records, and death register records.	Questionnaire	Age and sex	All PPIs except Rabeprazole, Pantoprazole	NR	10.9-11.2 years
Nolde et al.	2021	Germany	RS	No	6097740	1143948	35-65 (61.1)	44.10%	Hospital discharge diagnosis	Previous prescriptions	Age, sex and nationality	All PPIs	Regular use	6.2 years
Rooney et al.	2021	United States	RS	No	4436	1067	70-80 (75)	37%	Incident stroke	Questionnaire	Age, Sex, Race, education, smoking status, drinking status, physical activity index score, obesity	All PPIs	Regular use	5 years
Schmilovitz-weiss	2021	Israel	RS	No	29639	8600	65-95 (82.2)	38%	Brain computed tomography or magnetic resonance imaging during admission	Previous prescriptions	Age, gender and cardiovascular disease related risk factors	All PPIs	Regular use	1-7 years
Yang et al.	2021	China	PS	No	492479	49135	50-70 (60.0)	45.70%	Hospital discharge diagnosis or death registry	Verbal interview with touchscreen questionaire	Age, household income, hypertension, diabetes mellitus, total cholesterol, body mass index, alcohol consumption, physical activity, smoking habit, proton pump	All PPIs	Regular use	8 years
Sehested et al.	2018	Denmark	RS	No	7916	NR	44-66 (55)	43.30%	Hospital discharge diagnosis or death registry	Previous prescriptions	Age, sex, comorbidities and concomitant medication	All PPIs except Rabeprazole	Regular use	5.8 years
Nguyen et al.	2017	United States	PS	No	142489	425	30-75 (66.9)	35%	Incident stroke	Questionnaire	Age, sex, comorbidities and concomitant medication,indications for PPI use, including history of peptic ulcer disease, gastroesophageal reflux disease, or gastrointestinal bleeding,	All PPIs	On Prescription	12 years
Wang et al.	2017	Taiwan	RS	Yes	198148	15378	35-65 (51.7)	53.60%	Hospital discharge diagnosis or death registry	Previous prescriptions of 1 year	Age, sex, comorbidities, and concomitant medication	All PPIs	On Prescription	1 year

**Table-II:** Quality of included studies assessed using NOS criteria.

Study	Year	Selection				Comparability		Outcome		Total

		Representativeness of the exposed cohort	Selection of the nonexposed cohort	Ascertainment of exposure	Demonstration that outcome of interest	Basis of the design or analysis	Assessment of outcome	follow-up long enough for outcomes	Adequate follow up	
Kim et al.(21)	2022	1	1	1	1	1	1	1	1	8
Ma et al. (23)	2022	1	1	1	1	1	1	1	1	8
Park et al.(22)	2022	1	1	1	1	1	1	1	1	8
Bell et al.(25)	2021	1	1	0	1	1	1	1	1	7
Geng et al.(24)	2021	1	1	1	1	1	1	1	1	8
Nolde et al.(26)	2021	1	1	1	1	1	1	1	1	8
Rooney et al.(27)	2021	1	1	1	1	1	1	1	1	8
Schmilovitz-weiss et al. (28)	2021	1	1	1	1	1	1	1	1	8
Yang et al. (15)	2021	1	1	1	1	1	1	1	1	8
Sehested et al. (17)	2018	1	1	1	1	2	1	1	1	9
Nguyen et al.(16)	2017	1	1	1	1	1	1	1	1	8
Wang et al.(29)	2017	1	1	1	1	1	1	1	1	8

### Meta-analysis:

**Fig.2:**
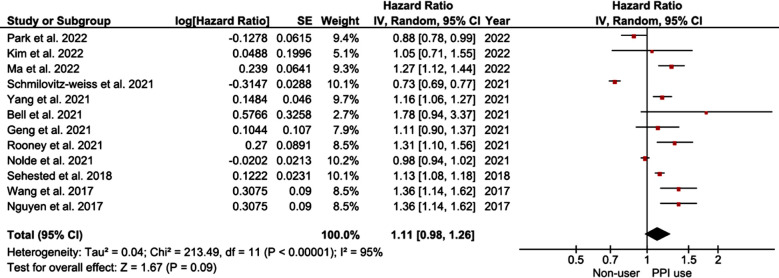
Forest Plot showing the cumulative effect estimate of hazard ratios for risk of stroke among PPI users and Non-users.

Among 12 included studies, the pooled hazard ratio (HR) for the overall risk of ischemic stroke on PPI use was 1.11, with a 95% CI ranging from 0.98 to 1.26, with p=0.09, indicating that the association was not statistically significant. However, there was considerable heterogeneity among the studies, as indicated by the I2 value of 95%. Fig.2

### Subgroup Analysis:

As shown in Table-III, when age was considered as a subgroup, PPI use correlated with significantly increased incidence of ischemic stroke among younger patients (<65 years old). The HR in this age group was 1.25, with a 95% CI of 1.07 to 1.45 (p=0.005). In patients > 65, PPI use still correlated with a slightly increased risk of ischemic stroke, with an HR of 1.09, a 95% CI of 1.01 to 1.18 (p=0.04). Both age subgroups exhibited some level of heterogeneity, with I2 values of 72% and 83%, respectively. Subgroup analysis based on the gender of patients showed higher risk of ischemic stroke in both male and female patients taking PPI. In males, the HR was 1.12 (95% CI: 1.02-1.24; (p= 0.01) and an I2 value of 77%. Among females, the HR was 1.21 (95% CI: 1.10-1.33; p<0.001) and an I2 value of 69%.

**Table-III:** Subgroup analysis of cumulative effect estimates of hazard ratios for risk of stroke among PPI users and non-users.

Sub-group		Studies	HR	CI	P value	I^2^ value
Overall Risk of Ischaemic stroke on PPI use		12	1.11	0.98-1.26	0.09	95%
Age	<65 years	6	1.25	1.07-1.45	0.005	72%
	>65 years	7	1.09	1.01-1.18	0.04	83%
Gender	Male	6	1.12	1.02-1.24	0.01	77%
	Female	5	1.21	1.10-1.33	<0.001	69%
Median Follow-up	Long term >5year	7	1.09	0.92-1.29	0.30	97%
	Short term≤5 year	5	1.14	0.96-1.35	0.13	83%
Type of PPI	Omeprazole	4	1.22	1.13-1.32	<0.001	16%
	Lansoprazole	4	1.13	1.05-1.22	0.0009	0%
	Esomeprazole	4	1.44	1.02-2.03	0.04	75%
	Pantoprazole	3	1.66	1.43-1.93	<0.001	0%
	Rabeprazole	2	1.07	0.70-1.62	0.76	39%
Frequency of PPI use	On Prescription	2	1.18	1.07-1.42	0.26	79%
	Regular use	7	1.08	1.01-1.19	0.13	83%
Propensity Score Matched	Yes	3	1.21	1.03-1.42	0.02	28%
	No	9	1.09	0.95-1.25	0.22	96%

The subgroup analysis based on the median follow-up duration showed that the risk of ischemic stroke associated with PPI use did not significantly differ between long-term (>5 years) and short-term (<5 years) follow-ups. The HR for the long-term follow-up group was 1.09 (95% CI: 0.92-1.29), p=0.30 and an I2 = 97%. Short-term follow-up group had HR of 1.14 (95% CI: 0.96-1.35), p=0.13 and an I2 = 83%.

In terms of the type of PPI used, the risk of stroke varied depending on the particular PPI used. Omeprazole and lansoprazole were associated with increased risks, with HRs of 1.22 (95% CI: 1.13-1.32) and 1.13 (95% CI: 1.05-1.22), respectively. Esomeprazole correlated with higher HR of 1.44 (95% CI: 1.02-2.03), while pantoprazole use had the highest HR of 1.66 (95% CI: 1.43-1.93). On the other hand, rabeprazole use was not specifically associated with higher ischemic stroke risk. Notably, the heterogeneity among studies varied for different PPIs.

In terms of the frequency of PPI use, subgroup analysis showed that both regular PPI use and PPI use as prescribed correlated with a slightly increased ischemic stroke risk, with HRs of 1.18 (95% CI: 1.07-1.42) and 1.08 (95% CI: 1.01-1.19), respectively. However, these associations were statistically significant, with substantial heterogeneity.

**Fig.3:**
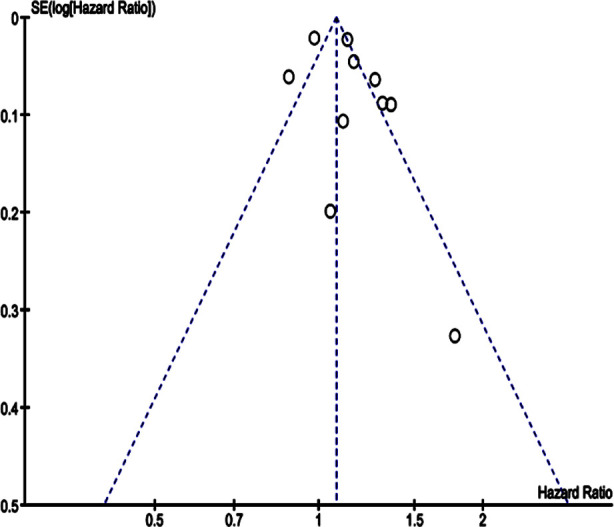
Funnel Plot showing the publication bias among studies showing estimate of hazard ratios for risk of stroke among PPI users and Non-users.

Lastly, the subgroup analysis based on propensity score matching (PSM) showed PSM did not markedly influence the association, with HR 1.21 (95% CI: 1.03-1.42) p= 0.02 and I2=28%, for the PSM subgroup of studies was, and the HR of 1.09 (95% CI: 0.95-1.25) p= 0.22 and an I2 value of 96% for the non-PSM subgroup. The funnel plot of the cumulative effect estimate of hazard ratios for risk of stroke among PPI users and non-users shows no publication bias for overall effect and sub-groups. Fig.3

### Sensitivity Analysis:

By removing studies one by one from the forest plot we demonstrated that the removal of the study by Schmilovitz-Weiss et al. 2021 changed the cumulative effect estimate to HR 1.15 95% CI [1.06,1.25], p=0.001, i2=85%, suggesting a potential effect of the study on the main results. However, the overall risk has to be considered with caution due to high heterogeneity.

## DISCUSSION

Our results showed that PPI use correlates with the increased risk of ischemic stroke, especially in individuals <65 years old, and this association is significant for all genders. Risk of stroke varies depending on the type of PPI used. A cumulative analysis of 12 included studies showed no significant correlation between PPI use and the risk of ischemic stroke, with a pooled HR of 1.11 and a 95% CI of 0.98-1.26. However, a significant association was found by the subgroup analysis. Our results are in agreement with the systematic review by Nolde et al.^18^ that included a total of five estimates and found no significant link between PPI use and the incidence of stroke, with a pooled HR of 1.08 95% CI (0.97, 1.20), p=0.111. However, they did not perform the subgroup analyses to identify any confounders. It is important to note that there was a considerable heterogeneity among the included studies in our review, as indicated by the high I2 value of 95%. This heterogeneity may be attributed to variations in study populations, study designs, follow-up durations, and PPI types across the studies.

Several studies have demonstrated that PPI use may lead to impaired endothelial function, which manifests in the reduced ability of blood vessels to dilate and regulate blood flow.^30^,^31^ Endothelial dysfunction plays a crucial role as an initial stage in the progression of atherosclerosis which is associated with elevated risk of heart attack and stroke.^30^ PPIs may also affect platelet function, potentially leading to increased platelet aggregation and clot formation.^32^ Additionally, PPI use has been associated with alterations in magnesium and calcium levels.^33^,^34^ Magnesium plays a vital role in maintaining cardiovascular health, and hypomagnesemia (low magnesium levels) has been reported in some patients using PPIs.^35^ Disturbances in calcium homeostasis can also impact cardiac function and increase the risk of cardiovascular events.^36^ Chronic use of PPIs has been linked to increased levels of inflammatory markers in the body,^37^ which is also linked to cardiovascular diseases, including atherosclerosis.

The results of our review showed that age did not appear to significantly impact the correlation between PPIs and the risk of stroke that was overall increased in patients who took PPI. However, this increased risk was much more substantial in patients < 65 (HR: 1.25 95% CI: 1.07-1.45), while in the age group >65 this risk was not as high (HR: 1.09 95% CI: 1.01-1.18). The analysis of gender as a potential effect modifier also did not show a significant impact. On the sub-group analysis, both male and female patients who took PPI had higher risk of stroke. The increased risk observed in both age and genders suggests a likely link between PPI use and stroke.

However, it is important to note relatively small number of studies available for each subgroup, which may have reduced the statistical power of the analysis. Additional studies with bigger sample sizes and more balanced gender and age representation are needed to validate our findings and provide in-depth understanding of potential age- and gender-related differences. Furthermore, the type of PPI used seemed to influence the risk of ischemic stroke. Omeprazole and lansoprazole showed a modestly increased risk, while esomeprazole exhibited a higher HR, suggesting a potentially stronger association with ischemic stroke. Notably, pantoprazole use correlated with the highest risk, while rabeprazole use had no significant association.

Our results further stress the importance of considering the specific PPI type when assessing the potential risks of stroke associated with PPI use. The observed discrepancy in the correlation between different PPI types and stroke risk could be attributed to several factors. Firstly, variations in the pharmacokinetic and pharmacodynamic properties of different PPIs may contribute to differential effects on cardiovascular health. For example, some PPIs like omeprazole may have a more profound impact on platelet aggregation or endothelial function, which are known to influence the risk of ischemic stroke. Pantoprazole was found to have a stronger link with ischemic stroke than omeprazole and lansoprazole.^17^

However, pantoprazole was given at higher doses, and this difference disappeared when the dosage was equivalent to that of other PPIs. Therefore, there is a possibility that the observed effect is dose dependent. Subgroup analysis based on follow-up duration demonstrated that the risk of ischemic stroke did not significantly differ between long-term (follow-up >5 years) and short-term (follow-up <5 years) studies. The duration of PPI use, therefore, may not substantially influence the risk of ischemic stroke.

### Limitation:

First, the included studies varied in terms of study design, population characteristics, follow-up duration, dose, and frequency of PPI use, which may have contributed to heterogeneity and potential biases. The differences in patient populations and study designs across the included studies may have influenced the observed links. Variation in patient characteristics, such as comorbidities or concomitant medication use, could potentially confound the observed association. Moreover, differences in study methodologies, including sample sizes and follow-up durations, may have affected the statistical power to detect significant links. Secondly, the included studies predominantly focused on patients without a history of cardiovascular diseases, which limits generalizability. Moreover, retrospective studies were included along with prospective ones, which could possibly add up to the selection bias.

## CONCLUSION

PPI use may be linked to increased risk of ischemic stroke, particularly in individuals below 65, both males and females. The risk of stroke may be impacted by the specific PPI type. However, no significant correlation was found on the overall analysis, and substantial heterogeneity was observed. These findings emphasize the importance of considering individual characteristics, such as age, gender, and PPI type, when assessing the potential risk of ischemic stroke is association with the use of proton pump inhibitors. Future studies are needed to further explore the underlying mechanisms and to validate our findings in diverse populations. Clinicians should carefully assess the potential risks and benefits of PPI use, especially in patients at higher risk of ischemic stroke.

### Authors’ contributions:

**YF:** Contributed to the design and manuscript writing.

**YF** and **YZ:** Contributed to the data collection and data analysis.

**YF:** Was involved in the critical revision of the manuscript.

All authors have read and approved the final manuscript.
